# Fingerprint Pattern Analysis for the Early Detection of Oral Potentially Malignant Disorders: A Cross-Sectional Study

**DOI:** 10.7759/cureus.91267

**Published:** 2025-08-29

**Authors:** Rritam Ghosh, Kanad Chaudhuri, Debasree Boral, Debanti Giri, Sayan Chattopadhyay, Keerthi K Nair, Rachita Arora

**Affiliations:** 1 Oral Medicine and Radiology, Dr. R Ahmed Dental College and Hospital, Kolkata, IND; 2 Dentistry, JIS School of Medical Science and Research, Kolkata, IND; 3 Pediatric and Preventive Dentistry, Dr. R Ahmed Dental College and Hospital, Kolkata, IND

**Keywords:** arch fingerprint, compound fingerprint, dermatoglyphics, oral precancer, radial loop, ulnar loop, whorl

## Abstract

Objective:Most oral cancers develop either de novo or from precancerous lesions such as leukoplakia, oral lichen planus (OLP), oral submucous fibrosis (OSMF), etc. This study aimed to investigate whether specific fingerprint patterns are associated with an increased risk of developing oral precancerous conditions.

Materials and methods: The study comprised 100 patients, of whom 50 healthy individuals of either sex were included as controls in group A, and 50 clinically and histopathologically proven oral precancer patients of either sex were included in group B. The fingerprints of 10 fingers from each individual were recorded via the Fingkey Hamster HFDU06 (Nitgen, Seoul, KOR) fingerprint scanner and qualitatively analyzed by the Nitgen Fingerprint Detector Software. The patterns were divided into five groups, namely arch, ulnar loop, radial loop, whorl, and compound. Comparison of the mean values among the study groups was carried out using the unpaired Student's t-test and one-way ANOVA.

Results: The comparison of the fingerprint patterns in each group was found to be statistically highly significant (p=0.000). The comparison of each fingerprint pattern with different grades of dysplasia of precancerous patients was not statistically significant (p˃0.05).

Conclusion: In the qualitative analysis of fingerprints, the whorl type of fingerprint pattern was the most prevalent in the control group, and the ulnar loop type was most prevalent in the oral precancer group. Thus, the ulnar loop pattern may be associated with higher risks of oral precancer irrespective of age or type of addiction.

## Introduction

Most oral cancers develop either de novo or from precancerous lesions such as leukoplakia, oral lichen planus (OLP), and oral submucous fibrosis (OSMF). According to estimates, the tendency of those with leukoplakia to develop carcinoma is greater than that of those with OSMF [1-3]. Several studies have suggested that OLP, especially the erosive form, may be associated with increased cancer risk. However, other investigators have questioned the strength of this association [2]. Thus, early detection is crucial to improve prognosis screening and enable management, saving millions of lives every year [4,5]. Precancer and cancer can be detected early using a variety of contemporary methods, such as ABO blood groups, palmar and sole prints, and fingerprints [5-7]. All primates have ridged palms and soles. Dermatoglyphics is the study of ridged patterns on the skin. It is a relatively new science that studies the fine-patterned ridges that run from digits to palms to soles of all primates. Cummins and Midlo (1926) coined the term 'dermatoglyphic', where derma means skin and glyphic means carvings [8]. In the course of a lifetime, fingerprint patterns remain unaffected by any severity of disease or condition, as stated by Cherrill [8]. 

During the 17 to 24 weeks of pregnancy, most dermatoglyphic traits are formed and remain unchanged throughout life [5]. The finger and palmar prints are permanent features, variable, and inherited by an individual. Fingerprint patterns differ among parents and their children, siblings, and even monozygotic, identical twins [9].

Keeping all of these factors in mind, the aim of the study was to analyze the digital dermatoglyphic patterns of patients with oral potentially malignant disorders (OPMD) and to correlate any type of specific fingerprint pattern with the risk of development of OPMD. The study also evaluates the predominance of fingerprint patterns per the grades of dysplasia and compares each pattern in the control and study groups. This study also evaluates the predominance of and compares each fingerprint pattern per the grades of dysplasia in the control and study groups.

## Materials and methods

Study design

The present study was conducted in the Department of Oral Medicine and Radiology at Dr. R Ahmed Dental College and Hospital, Kolkata, India, after obtaining approval from the Institutional Ethics Committee (approval no. RADCH/EC/79/2024). Patients with OPMD, preferably oral leukoplakia and OSMF (clinically and histologically diagnosed), and with or without habits such as smoking or chewing tobacco, were included in the study group. For the control group, we chose healthy individuals free from OPMD and any habit of smoking or chewing tobacco. The exclusion criteria were persons with congenital or acquired deformities of fingers and fingerprint patterns that are not readable in a patient with adermatoglyphia; bee stings; scars or wounds; plastic surgery; and burns or cuts on fingertips.

Sample size calculation

The minimum sample size for each group was calculated using the two-sample T-interval, as the data values are independent and sampled from two populations. The two independent groups have equal variances. The sample size n is calculated using the following formula:

n = (Zα/2+Zβ)2 2σ2 / d2

The critical value Zα/2 refers to the point in the normal distribution associated with α/2. For instance, with a confidence level of 95%, α is set at 0.05, resulting in a critical value of 1.96. Similarly, Zβ is the critical value corresponding to β. For a power of 80%, β equals 0.2, making the critical value 0.84. Additionally, σ² represents the population variance, which is 75 in our study, and d denotes the difference we aim to detect (i.e., 5).

According to this formula, the sample size was 50 for the study and control groups. Informed consent was taken from all patients participating in the study, having stated clearly that the collected data would be solely used for research purposes and full confidentiality would be maintained. Group A included 50 healthy individuals of either sex, clinically free from oral precancer and cancer, who were considered the control group. Group B had 50 patients of either sex who had been clinically and histologically diagnosed with an OPMD, especially oral leukoplakia and OSMF. All the results were entered into Microsoft Excel (Microsoft Corp., Redmond, WA, USA) and analyzed using SPSS Statistics version 27.0 (IBM Corp., Armonk, NY, USA). 

Methodology

**Figure 1:**
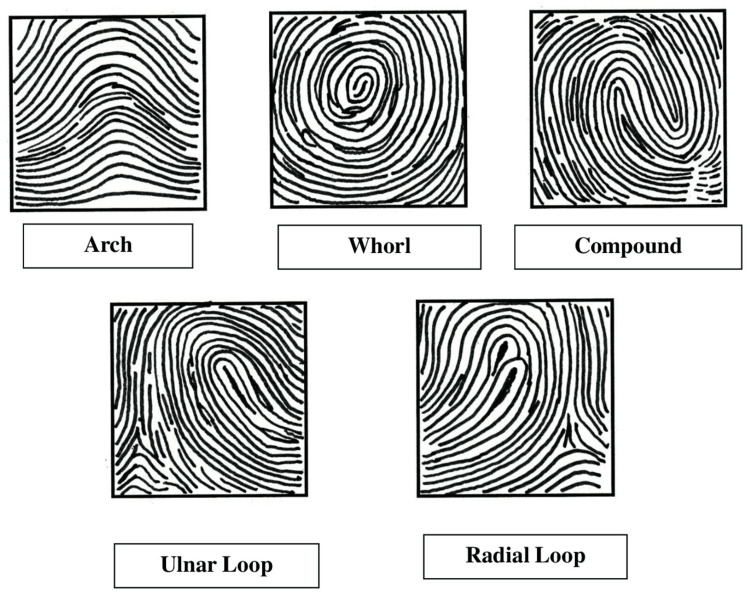
Schematic diagrams of different fingerprint patterns Figure reused from *Digital Dermatoglyphics — A New Approach in Early Detection of Oral Cancer* by Ghosh and Barman [5], with a Creative Commons Attribution 3.0 license (CC BY).

To enhance the quality of dermatoglyphic prints, sweat, oil, and dust were removed from the skin on the fingertips of the subjects. This was done by washing the ridged areas with soap and water and drying them. Using the Fingkey Hamster HFDU06 (Nitgen, Seoul, KOR) fingerprint scanner, all 10 fingers were simultaneously placed over its scanning surface. The fingerprints were then transferred to a computer via USB and processed using the Nitgen Fingerprint Detector Software. The images are then saved as a bitmap image (BMP). We followed the fingerprint scanning method proposed by Batchelor [10]. Based on the classification given by Galton (1892) and modified by Henry (1937), fingerprint images are divided into five groups: arch, ulnar loop, radial loop, whorl, and compound (Figure 1) [8,11].

## Results

The study consisted of 100 subjects, comprising 50 controls and 50 clinically and histologically proven OPMDs. In the control group, the mean age of participants was 22 years, with 36 male participants and 14 female participants. In the study group, 43 male participants and only seven female participants were recorded, with the mean age of participants being 28 years. Among 50 precancerous patients, 27 (54%) belonged to homogeneous leukoplakia and 23 (46%) belonged to OSMF. The number of homogeneous leukoplakia cases was higher in our study than in OSMF (Table 1).

**Table 1:** Distribution of precancerous patients in group B OSMF: Oral submucous fibrosis

Group	Type of lesion		Total n
Group B	Homogeneous leukoplakia	OSMF	
	27 (54%)	23 (46%)	50

In group A, the fingerprint pattern that was most common was the whorl pattern (273, 54.6%), whereas the minimum was the radial loop (six, 1.2%). In group B, the fingerprint pattern with the maximum number was the ulnar loop (235, 47%), whereas the minimum was the radial loop (four, 0.8%) (Table 2). The comparison of fingerprint patterns between group A and group B showed a highly significant difference (p<0.05) between the ulnar loop, whorl, and compound (Table 3). A comparison of each fingerprint pattern with different grades of dysplasia of precancerous patients showed no statistically significant difference (p>0.05), with no correlation existing between fingerprint patterns and grades of dysplasia in precancerous patients (Table 4).

**Table 2:** The type of fingerprint patterns found among the two groups

Groups	Arch		Ulnar loop		Radial loop		Whorl		Compound	
	Number (n)	Mean ± SD	Number (n)	Mean ± SD	Number (n)	Mean ± SD	Number (n)	Mean ± SD	Number (n)	Mean ± SD
Group A (controls)	26 (5.2%)	0.89 ±0.51	171(34.2%)	3.41 ±2.90	6(1.2%)	0.42 ±0.11	273(54.6%)	5.45 ±3.33	24(4.8%)	0.98 ±0.52
Group B (study)	34(6.8%)	1.75 ±0.68	235(47%)	4.70 ±2.53	4(0.8%)	0.34 ±0.08	151(30.2%)	3.02 ±2.46	76(15.2%)	1.62 ±1.52

**Table 3:** Comparison of each fingerprint pattern between groups A and B NS: Not significant, HS: Highly significant

Values	Arch		Ulnar loop		Radial loop		Whorl		Compound	
	Group A	Group B	Group A	Group B	Group A	Group B	Group A	Group B	Group A	Group B
Total	26	34	171	235	6	4	273	151	24	76
Mean±SD	0.89 ±0.51	1.75 ±0.68	3.41 ±2.90	4.70±2.53	0.42 ±0.11	0.34 ±0.08	5.45 ±3.33	3.02±2.46	0.98 ±0.52	1.62 ±1.52
p-value	0.951 (NS)		0.002 (HS)		0.403 (NS)		0.000 (HS)		0.003 (HS)	

**Table 4:** : Distribution and comparison of each fingerprint pattern among different grades of dysplasia in group B NS: Not significant

Type of fingerprints	Grades of dysplasia								F-value	p-value
	0		I		II		III			
	Number (n)	Mean ± SD	Number (n)	Mean ± SD	Number (n)	Mean ± SD	Number (n)	Mean ± SD		
Arch	7	1.64±0.78	9	2.06±0.60	5	0.57±0.28	13	2.77±1.63	1.116	0.352 (NS)
Ulnar loop	35	3.89±2.57	76	5.07±2.31	76	4.22±2.69	48	6.00±2.33	1.356	0.267 (NS)
Radial loop	0	0.00±0.00	2	0.35±0.13	2	0.47±0.11	0	0.00±0.00	0.469	0.704 (NS)
Whorl	34	3.78±3.07	43	2.87±1.96	63	3.50±2.68	11	1.41±1.38	0.522	0.465 (NS)
Compound	14	1.67±1.56	20	1.35±1.33	34	1.97±1.89	8	1.20±1.00	0.639	0.593 (NS)

## Discussion

Amongst various OPMD, oral leukoplakia and OSMF have been shown to have a high rate of transformation to oral cancer. Multiple studies have shown that the prevalence of OSMF in India varies between 0.03% and 3.2% [12,13]. Tobacco is considered to be one of the main risk factors for oral cancer among youths [14].

The study group consisted of 50 subjects with clinically and histopathologically diagnosed OPMDs. In our study, the majority of patients had homogeneous leukoplakia and OSMF since these two are the most common potentially malignant oral disorders in the Indian population, as stated by Rajendran [15]. In this study group, homogeneous leukoplakia cases were more common (54%) than OSMF (46%), since leukoplakia is the most common oral premalignant lesion, as described by Osuna and Hopkins [16].

A certain percentage of the population cannot reverse DNA damage, and precancerous or cancerous lesions tend to damage the DNA. As a result, researchers are always looking for genetic markers that can be used to diagnose oral cancer [5]. Many early cancer detection methods exist, but dermatoglyphics is noninvasive and easy to perform [17]. According to Cherrill, fingerprints remain immutable for a lifetime, regardless of the severity of the disease's condition [8]. Some studies have been undertaken using dermatoglyphics as indicators of congenital heart disease, leukemia, cancer, celiac disease, intestinal disorders, rubella embryopathy, Alzheimer's disease, schizophrenia, and other forms of mental illness [6]. Unusual fingerprint patterns may indicate gene or chromosomal abnormalities associated with diseases like oral precancer and cancer [5,18].

This study aimed to reveal the early diagnosis of oral precancers by qualitatively comparing fingerprint patterns. A comparison of fingerprint patterns between patients with OPMD and healthy individuals was made to identify the high-risk patients so as to help undertake early preventive measures in those susceptible individuals. Furthermore, fingerprint patterns in oral precancer patients were correlated with their degree of dysplasia [5].

The predominant fingerprint pattern in the control group was the whorl (54.6%, 273), and the minimum was the radial loop (1.2%). Our observation is consistent with studies conducted by Venkatesh et al. and Agarwal et al., who stated that the whorl-type fingerprint pattern was most predominant in the healthy population [18,19]. Similarly, in a study conducted by Darna et al., it was found that the whorl pattern was the most dominant in the control group, and this finding was also similar to our study [20].

In our study, fingerprint patterns in the precancer/study group showed a high prevalence of the ulnar loop (47%, 235), which is per the study conducted in the year 2008 by Venkatesh et al. [18]. The radial loop was less prevalent (four, 0.8%), which is per the study conducted by Agarwal et al. [19]. Additionally, a reduced arch and whorl pattern was noted in the study group, and this observation aligns with findings from Dutta et al. [21].

A study conducted by David and Sinha on patients with pre-malignant diseases (PMDs), oral squamous cell carcinoma (OSCC), and with/without harmful habits but no visible lesions found that the loop pattern was the most common in the PMD group and the whorl pattern in the control group. However, there was a decreased frequency of arch patterns across all study groups. All of these findings [22] align with the results of our study.

Gupta A and Karjodkar FR found that patients with OSMF exhibited more arch and ulnar loop fingerprint patterns, while simple whorl patterns decreased, aligning with our study [23]. A separate study by Vaishali et al. in Ahmedabad revealed that loop patterns were the most common, occurring in 54.5% to 69.5% of cases, followed by whorl patterns, which ranged from 28.5% to 42.5% in patients with OLP and OSMF [24].

Studies by Deepa et al. and Singh et al. showed increased arch patterns in PMDs, contradicting our study results, and this result proves that the type of fingerprints associated with precancerous lesions and conditions differs with different populations [25,26]. A study reported by Fuller showed that several genes control the development of finger and palm dermatoglyphics, which also predispose individuals to premalignancy and malignancy [27]. We found statistically significant results while comparing the fingerprint patterns in both groups (p≤0.05). It shows the variability, uniqueness, and individuality of fingerprint patterns in West Bengal. Cummins established the variability of fingerprint patterns among different races in his studies conducted between 1926 and 1967 [28]. Gilligan et al. suggested a significant correlation between dermatoglyphics and geographic areas, confirming the biological validity of social and ethnic criteria [29].

Our study found statistically non-significant relationships (p > 0.05) between fingerprint patterns and different grades of dysplasia within the precancer group. This indicates no correlation between fingerprint patterns and the grades of dysplasia in oral precancerous lesions.

A few of the limitations of our study are the small sample size, participation of only one population from one geographic area, and inclusion of only patients with OPMDs. Confounders such as alcohol use, genetics, or family history should also be considered. For future studies, our recommendations are the inclusion of other OPMDs like OLP or erythroplakia for broader applicability; varied populations; multiple geographic areas, and, most importantly, establishing a definitive genetic link between fingerprints and OPMD.

## Conclusions

Per our qualitative analysis of fingerprint patterns, the differences between each type were significant, thus proving the uniqueness of fingerprints. We can conclude that the connection between fingerprint patterns and oral precancerous lesions (or any cancer) lies in their genetic dependence on development. It is suggested that numerous genes involved in finger and palmar dermatoglyphic development may also indicate premalignancy.
